# Long-term evaluation of outcomes and costs of urolithiasis re-interventions after ureteroscopy, extracorporeal shockwave lithotripsy and percutaneous nephrolithotomy based on German health insurance claims data

**DOI:** 10.1007/s00345-022-04180-3

**Published:** 2022-10-14

**Authors:** Claudia Konnopka, Benedikt Becker, Christopher Netsch, Thomas R. W. Herrmann, Andreas J. Gross, Lukas Lusuardi, Thomas Knoll, Hans-Helmut König

**Affiliations:** 1 Department of Health Economics and Health Services Research, University Medical Center Hamburg-Eppendorf, Martinistr. 52, 20246 Hamburg, Germany; 2 Department of Urology, Asklepios Hospital Barmbek, Hamburg, Germany; 3 Department of Urology, Spital Thurgau AG, Kantonsspital Frauenfeld, Frauenfeld, Switzerland; 4 Department of Urology and Andrology, Salzburg University Hospital, Paracelsus Medical University, Salzburg, Austria; 5 Department of Urology, Klinikum Sindelfingen-Boeblingen, Sindelfingen, Germany

**Keywords:** Urolithiasis, Healthcare costs, Sick leave, Administrative claims, Health care

## Abstract

**Purpose:**

Comparisons of ureteroscopy (URS), extracorporeal shockwave lithotripsy (SWL) and percutaneous nephrolithotomy (PCNL) for urolithiasis considering long-term follow-up are rare. We aimed to analyze re-intervention rates, costs and sick leave days of URS, SWL and PCNL patients within 7 years.

**Methods:**

This retrospective cohort study was based on German health insurance claims data. We included 54,609 urolithiasis patients incidentally treated in 2008–2010. We investigated time to re-intervention, number of sick leave days and healthcare costs. We applied negative binomial, extended Cox regression and gamma models.

**Results:**

54% were incidentally treated with URS, 40% with SWL and 6% with PCNL. 15% of URS, 26% of SWL and 23% of PCNL patients were re-treated within 7 years. Time to re-intervention was significantly lower for PCNL (955 days) and SWL (937 days) than URS (1078 days) patients. Costs for incident treatment were significantly higher for PCNL (2760€) and lower for SWL (1342€) than URS (1334€) patients. Yet, total costs including re-interventions were significantly higher for PCNL (5783€) and SWL (3240€) than URS (2979€) patients. Total number of sick leave days was increased for PCNL (13.0 days) and SWL (10.1 days) compared to URS (6.8 days) patients.

**Conclusion:**

This study describes outcomes after use of different intervention options for urolithiasis. URS patients showed longest time free of re-interventions and lowest number of sick leave days. Although SWL patients initially had lower costs, URS patients had lower costs in the long run. PCNL patients showed high costs and sick leave days.

**Supplementary Information:**

The online version contains supplementary material available at 10.1007/s00345-022-04180-3.

## Introduction

Urolithiasis is a common disease with a prevalence of 5% in Germany [1]. The prevalence and incidence have been increasing in the last decades [1, 2]. Risk factors are, e.g., male sex, nutrition, lifestyle and physical activity [3], and comorbid conditions as obesity and diabetes mellitus [4, 5].

In Germany, urolithiasis treatments usually take place on an inpatient basis. Urolithiasis patients have to stay in hospital for 3.6 days on average [6]. Urolithiasis has a recurrence rate of 30–50% within 10 years [7] and primarily affects working-age adults [8]. Thus, urolithiasis causes high healthcare costs and productivity loss.

The three most common procedures for removing urinary tract stones are extracorporeal shockwave lithotripsy (SWL), ureteroscopy (URS) and percutaneous nephrolithotomy (PCNL). SWL has some contraindications and a modest stone-free rate [9, 10]. URS has little contraindications and a sufficient stone-free rate [9]. URS seems to be more cost-effective than SWL due to higher stone-free rates and lower costs [11–13]. There is a trend away from SWL and toward URS [14–16]. PCNL entails a high stone-free rate, but higher costs [9, 11].

Urolithiasis has a high recurrence rate when observed over several years. Yet, the existing literature mainly focused on short follow-up [13, 15, 17], due to lacking long-term data. To overcome this limitation, health insurance claims data can provide a long-term follow-up with a low attrition rate. Using these data, we aimed to investigate patients treated with URS, SWL and PCNL regarding re-interventions, healthcare costs and sick leave days within a 7-year follow-up.

## Material and methods

For this retrospective cohort study, data were provided by the Scientific Institute of the AOK (“Wissenschaftliches Institut der AOK”) (WIdO). The WIdO administers data of the largest association of statutory health insurance companies in Germany covering about one-third of the German population. Data were available for the years 2007–2017. Six months preceding index treatment as washout period and 7 years as follow-up were applied; thus, 06/2007–12/2010 was used as index period to identify incident urolithiasis patients. As anonymized claims data were used, informed consent of the study participants and approval of an ethics committee were not applicable.

All cases of patients insured by the AOK, living in Germany, with an incident urolithiasis diagnosis and treatment with URS, SWL or PCNL during index period were included. The codes used for identification can be found in Supplementary Table 1. We excluded cases with no incident urolithiasis treatment, i.e., cases with URS, SWL or PCNL treatment in the 6-month period preceding the index treatment, and combined treatments within the same day (*n* = 1305; 0.02%).

Re-intervention as proxy for the stone-free rate was measured as additional treatment of URS, SWL or PCNL after index hospital discharge. Sick leave days with an urolithiasis diagnosis were recorded in claims data. Urolithiasis-specific hospital costs were measured as index, re-intervention and total costs. They were inflated in 2016 Euro using the Gross Domestic Product price index [18, 19]. In Germany, hospital costs are calculated as fixed amount based on diagnosis-related groups per inpatient or outpatient hospital case, i.e., one amount for all treatments during one hospital stay. Apart from negligible co-payments, health insurances reimburse these costs to the hospitals. By using the corresponding claims data, we applied a payer perspective on costs. To avoid bias by extreme outliers, all costs were winsorized at the 99% percentile. We used sex, age at index date, year of index treatment, treatment setting (inpatient or outpatient), 4-digit index treatment ICD code as rough proxy for the stone localization and Elixhauser comorbidities based on the ICD-10 codes at index hospital admission [20, 21] for risk adjustment.

We compared patient characteristics descriptively between groups incidentally treated with URS, SWL and PCNL. We analyzed the number of re-interventions using generalized linear models (GLMs) with negative binomial distribution and log link function. Regarding time to re-intervention, we applied an extension of the Cox proportional hazard model [22], the Prentice, Williams and Peterson (PWP) gap time model with robust standard errors [23, 24]. Gap time was defined as the number of days from index or last to next treatment. We found no serious violations of the proportional hazards assumption [25, 26]. For healthcare costs, we used GLMs with gamma distribution and log link function, and for sick leave days, we used GLMs with negative binomial distribution and log link function. Cost and sick leave day results were reported as average marginal effects.

## Results

Of 54,609 urolithiasis patients, 29,441 (53.91%) were incidentally treated with URS, 21,848 (40.01%) with SWL and 3320 (6.08%) with PCNL (Table 1, Supplementary Table 2). Of URS and SWL patients, about two-thirds were male, whereas for PCNL patients, the share of males was lower. Patients were on average 52.6 years old, with PCNL patients slightly older.

**Table 1:** Descriptive patient characteristics

	Total		URS		SWL		PCNL	
*N*, %	54,609		29,441	53.91%	21,848	40.01%	3320	6.08%
Baseline characteristics (date of treatment)								
Age: mean, SD	52.6	16.2	51.7	16.6	53.3	15.6	56.3	15.8
Sex: *n*, %								
Female	19,086	35.0%	9697	32.9%	7843	35.9%	1546	46.6%
Male	35,523	65.0%	19,744	67.1%	14,005	64.1%	1,774	53.4%
Treatment setting: *n*, %								
Inpatient	54,535	99.9%	29,385	99.8%	21,830	99.9%	3320	100.0%
Outpatient	74	0.1%	56	0.2%	18	0.1%	–	0.0%
Follow-up within 7 years after index treatment								
Number of patients with re-interventions: *n*, %								
0	43,632	79.9%	24,950	84.7%	16,123	73.8%	2559	77.1%
1	7940	14.5%	3358	11.4%	4054	18.6%	528	15.9%
2	1995	3.7%	783	2.7%	1051	4.8%	161	4.8%
3 or more	1042	1.9%	350	1.2%	620	2.8%	72	2.2%
Time to first re-intervention, if occurring [days]: mean, SD	996	680.2	1078	685.7	937	670.9	955	668.0
Hospital costs (inpatient or outpatient) [EUR]: median, IQR								
For incident treatment	2263	1848–2812	2266	2093–2525	2060	1285–2839	4407	4082–5073
For re-interventions, if occurring	2385	1848–2812	2465	2033–3119	2276	1358–3050	2909	2159–4280
In total	2399	1848–2812	2308	2144–2954	2650	1737–3752	4632	4174–6877
Number of sick leave days: median, IQR								
After incident treatment	0	0–11	0	0–11	0	0–13	0	0–12
After re-interventions, if occurring	0	0–10	0	0–11	0	0–8	0	0–15
In total	0	0–10	0	0–10	0	0–10	0	0–10

*URS* ureteroscopy, *SWL* extracorporeal shockwave lithotripsy, *PCNL* percutaneous nephrolithotomy, *SD* standard deviation, *IQR* interquartile ratio

Of all patients, 20.1% had at least one re-interventions. This share was higher for patients incidentally treated with PCNL and particularly with SWL, and lower for those with URS. Time to re-intervention was shorter for PCNL and particularly SWL, and longer for URS patients. Median urolithiasis-specific hospital costs for the incident treatment were higher for URS than SWL patients, but total costs within follow-up were higher for SWL than URS patients. PCNL patients showed the highest median costs for both incident treatment and in total.

**Fig. 1:**
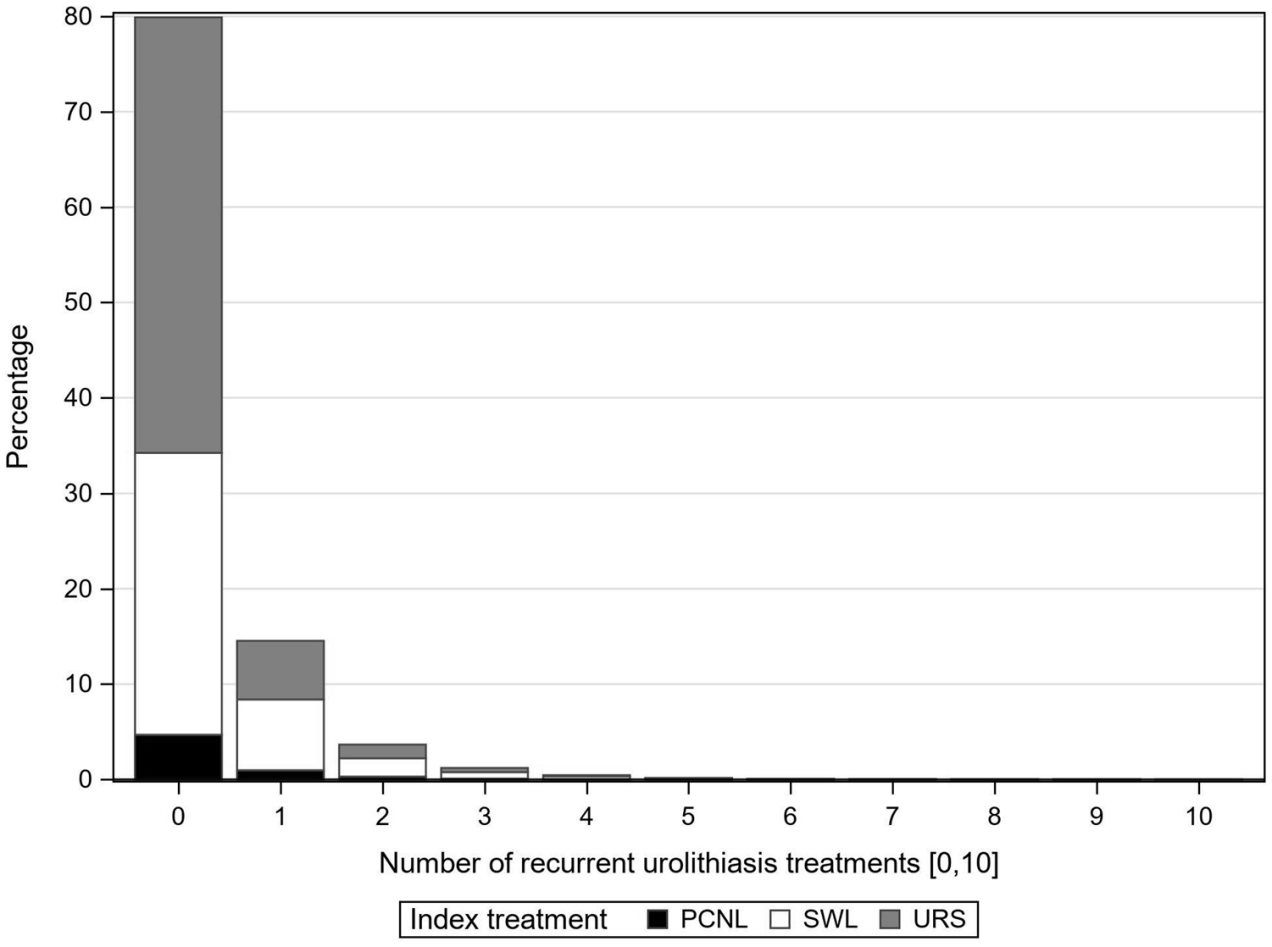
Proportion of patients with urolithiasis re-interventions, stratified by index treatment

Figure 1 presents the proportion of re-interventions for all urolithiasis patients. For patients with no re-intervention, the proportion of patients incidentally treated with URS was highest. For patients with one or more re-interventions, the proportion of index SWL patients increased. This stands in line with the high number of re-interventions for patients incidentally treated with SWL.

**Fig. 2:**
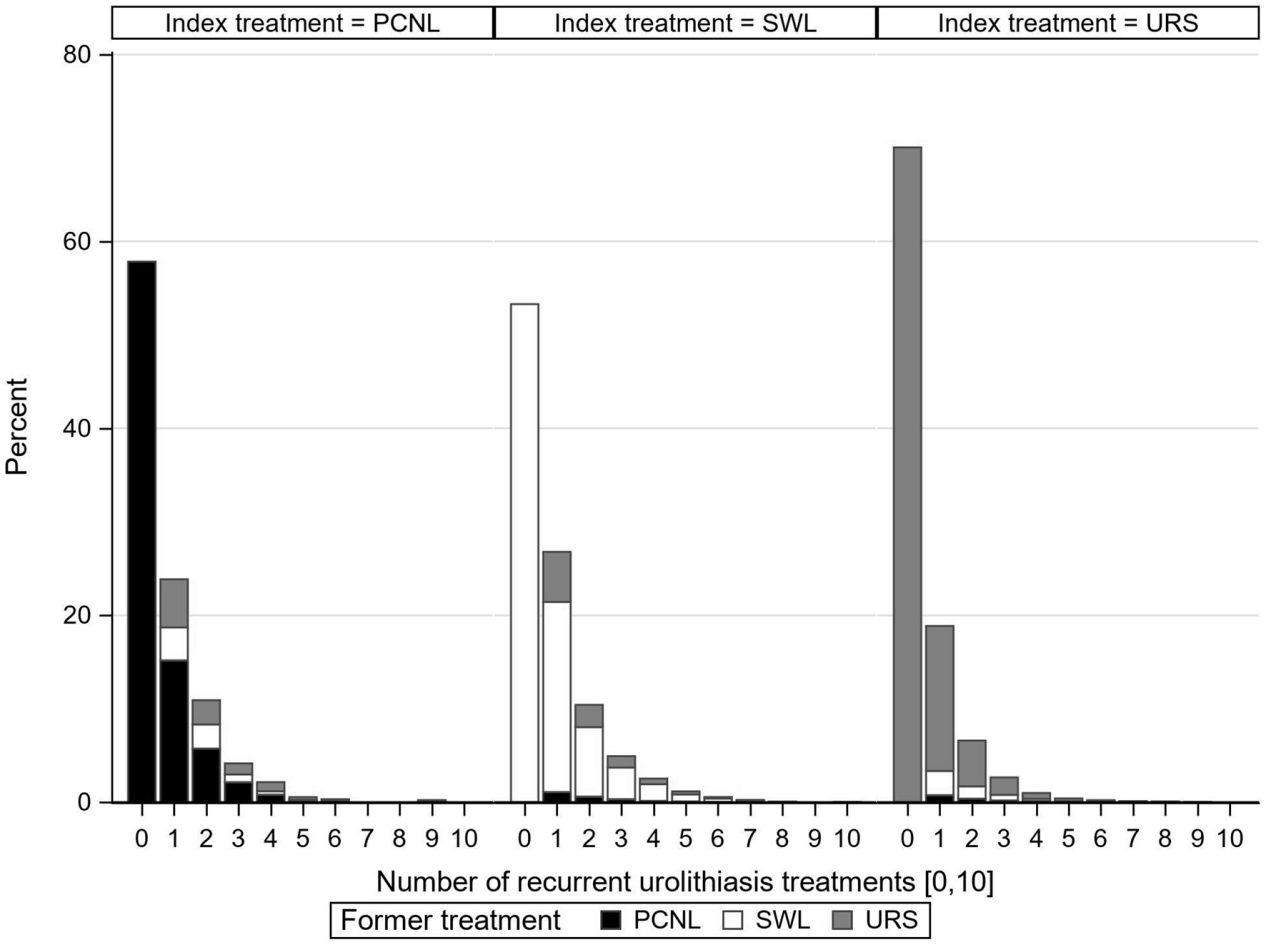
Proportion of cases with urolithiasis re-interventions, stratified by index and former treatment

Figure 2 displays the proportion of cases instead of patients (i.e., one patient can be counted as several cases, if treated repeatedly) to investigate treatment changes. Cases were stratified by patients’ index treatment. Particularly for patients with index treatment URS, the proportion of cases of one or re-intervention also with URS was remarkably higher than the proportion of cases repeatedly treated with PCNL or SWL. In other words, re-intervention after incident URS treatment was most often also done with URS. This also applied to index PCNL and SWL treatment. Thus, subsequent treatment options were most often chosen in line with the incident treatment option.

Patients incidentally treated with PCNL and particularly with SWL showed a significantly (*p* < 0.0001) higher risk of an increased number of re-interventions (Supplementary Table 3), which emphasizes descriptive results. The risk was significantly (*p* < 0.0001) increased for lower age, males and outpatient treatment setting.

Supplementary Table 4 shows model results estimating the time to re-interventions. The hazard for the first and any re-intervention was significantly (*p* < 0.0001) increased for patients incidentally treated with PCNL and particularly SWL, compared to URS. When adjusting for treatment change after the incident treatment, this significantly (*p* < 0.0001) affected the hazard ratio.

To investigate the interaction of treatment change and index treatment, we estimated risk scores, calculated as the estimated risk of a re-intervention for each index treatment option and changed or unchanged former treatment. All other variables were set to their mean. Supplementary Fig. 1 displays the estimated risk scores stratified by index treatment and former treatment change. Change of treatment was associated with an increased risk of an additional re-intervention for all index treatment options. While the risk for a re-intervention was higher for incident SWL than incident URS without treatment change, it was lower for incident SWL than incident URS with treatment change.

Patients incidentally treated with SWL were associated with significantly (*p* < 0.0001) lower urolithiasis-related healthcare costs regarding index treatment (average marginal effect (AME) = 1342€); however, in total they showed higher costs (AME = 3240€) than those incidentally treated with URS (AME = 1334 and 2979€) (Supplementary Table 5). PCNL patients showed significantly (*p* < 0.0001) higher costs both for index treatment (AME = 2760€) and over the total follow-up period (AME = 5783€).

Urolithiasis-related sick leave days were significantly (*p* < 0.0001) increased for index treatment and in total for SWL (AME = 7.1 and 10.1) and PCNL (AME = 9.8 and 13.0) patients, compared to URS (AME = 5.3 and 6.8) patients (Supplementary Table 6).

## Discussion

In this study, urolithiasis patients treated with URS, SWL and PCNL within a particularly long follow-up of 7 years were investigated. About 20% of all patients received re-intervention with one of the three treatments, with PCNL and particularly SWL patients having a higher risk than URS patients. If patients were treated repeatedly, the treatment method was often retained. If treatment was changed, it was more commonly changed from PCNL or SWL to URS than from URS to another option. After incident PCNL and SWL treatment, treatment change (e.g., re-intervention with URS) was associated with a decreased hazard of another treatment. This may be due to the decreased risk of re-intervention after URS treatment and due to the fact that a second-look surgery increases the stone-free rate.

These results stand in line with the literature [10] and emphasize the trend away from SWL and toward URS [14, 15]. However, for PCNL patients we found a slightly shorter time to and a larger number of re-interventions than for URS patients contradicting the existing literature [9], although this should be compared according to the stone size on which we have no information here. In terms of stone-free rates, PCNL is usually preferred over URS [27, 28]. Probably, in the analyzed database PCNL was conducted for cases with particularly large or complex stones, and these cases needed re-intervention more often than URS cases due to the complex nature of urolithiasis. Furthermore, we had no information on the treatment site of the re-intervention. Thus, some cases may have been additional treatments on the other side. It should be noted that re-intervention rates do not perfectly measure stone-free rates, but serve as a proxy. Therefore, the re-intervention rate we found was lower than in the literature [7].

URS cases showed lowest total healthcare costs, although the index hospital treatment was more expensive for URS than for SWL cases. Although SWL is usually applied for patients with small-size stones, the high costs may be caused by the high re-intervention rate for SWL cases which would entail higher total inpatient costs. However, costs depend on the country’s healthcare system. In Germany, all necessary treatments are reimbursed by the mandatory health insurance and out-of-pocket expenditures are neglectable. Furthermore, the majority of SWL patients were treated in an inpatient setting with higher costs as opposite to many other countries. Cost comparisons should consider these differences.

There is evidence that URS is more cost-effective than SWL with both higher stone-free rate and lower costs [11–13], although another study demonstrated lower costs for SWL [29]. In our study, PCNL patients had considerably higher costs. In the literature, two studies compared PCNL with SWL [30] and URS [31], and both showed higher costs and effectiveness for PCNL. Overall, the indications for the three treatment options differ, which limits the feasibility of health economic evaluations.

Economic considerations may affect the treatment choice. For example, purchasing a SWL device is very expensive and hospitals owning it may opt for SWL more often than others. On the other side, complex and more expensive PCNL may be conducted particularly in larger hospitals with a sufficient economy of scale.

Patients treated with URS had the lowest and patients with PCNL the highest number of sick leave days, both after index treatment, re-intervention and in total. This may be due to the degree of invasiveness of the treatment options. Particularly for PCNL and SWL patients, the number of sick leave days inclined when comparing the index and the total follow-up period, which could also be explained by the higher re-intervention rate.

This study has some limitations. Although we adjusted for various patient factors affecting the observed outcomes, they may not fully capture the patients’ risk profile. There are further risk factors, for example lifestyle or physical activity [3]. However, data on patient characteristics are only available to a limited degree in claims data. The quality of claims data may be limited, although they have a high validity due to their reimbursement purpose. Furthermore, patients may be selected for a treatment due to certain factors which cannot be observed in claims data, e.g., stone size and exact location. Therefore, a selection bias is likely. Thus, our results are not causal effects of the treatment, but rather correlations for patients for whom the treatment was chosen. Furthermore, the measurement based on claims data may not fully capture all information on, e.g., stone clearance rate or treatment site. Thus, we cannot distinguish between re-interventions on the same side and treatments on the other side. Moreover, the lack of information on stone burden limits the comparison of health and health-economic outcomes related to endourological procedures for treating urolithiasis.

Yet, our study adds to existing knowledge on the treatment of urolithiasis by drawing on a large and rich dataset of almost 55,000 patients allowing a follow-up of 7 years. We used claims data which are less vulnerable to, e.g., recall bias, an issue common for survey data. The AOK has a high national coverage of about one-third of the German population, which makes our results representative. To the best of our knowledge, this is the first comprehensive study comparing URS, SWL and PCNL for urolithiasis patients over such a long time horizon.

## Conclusion

This study provides insights into re-intervention rates, costs and sick leave days for urolithiasis patients over a period of 7 years. Patients incidentally treated with URS showed a relatively long time without re-interventions, few sick leave days and low total healthcare costs; those treated with SWL had lower initial costs, but higher total costs; and those with PCNL had the highest probability of re-interventions and increased healthcare costs and sick leave days.

## Supplementary Information

Below is the link to the electronic supplementary material.

Supplementary file1 (DOCX 105 KB)

## Data Availability

The datasets supporting the conclusions of this article are owned by the German statutory health insurance AOK. Since public deposition of the data would breach ethical and legal compliance, data are only available upon formal request from the research institute of the AOK (WIdO). To request the data, please contact the institutional body of the WIdO (wido@wido.bv.aok.de). In order to fulfill the legal requirements to obtain that kind of data, researchers must obtain a permission for a specific research question from the German Federal (Social) Insurance Office. Additionally, researchers must conclude a contract with the statutory health insurance regarding data access which can be requested from the "AOK-Bundesverband GbR" (Federal Association of Local Health Insurance Funds) under http://aok-bv.de/kontakt/. The licensee is permitted to use the data for the purpose of the research proposal within their company, exclusively. Thereby, company is defined as an economical unit. Licensees are not allowed to pass the data to a third party or to create software or databases with the exception of scientific publications. Moreover, the study has to be approved by the data protection officer at both the statutory health insurance and the research institute.

## Code availability

Code is available by request. Please contact the corresponding author.
