# Supplementary material for: Long-term evaluation of outcomes and costs of urolithiasis re-interventions after ureteroscopy, extracorporeal shockwave lithotripsy and percutaneous nephrolithotomy based on German health insurance claims data
Source: World J Urol. 2022 Oct 14;40(12):3021–7. doi: 10.1007/s00345-022-04180-3 (PMC9712271; doi:10.1007/s00345-022-04180-3)

## Supplement

**Supplementary table 1:** ICD- and OPS-codes for urolithiasis treatment or complications during or after treatment

| **Diagnosis or treatment** | **ICD- or OPS-Codes** |
| --- | --- |
| Urolithiasis | N20, N21, N22, N23 |
| Ureteroscopy | 5-562.4, 5-562.5, 5-562.8, 5-550.21, 5-550.31 |
| Extracorporeal shock wave lithotripsy | 8-110.1, 8-110.2 |
| Percutaneous nephrolithotomy | 5-550.20, 5-550.30, 5-562.6, 5-562.7 |
| Abbreviations: ICD = International Classification of Diseases; OPS = operation and procedure codes. | |

**Supplementary table 2**: Descriptive patient characteristics

|  | **Total** | | **URS** | | **SWL** | | **PCNL** | |
| --- | --- | --- | --- | --- | --- | --- | --- | --- |
| N, % | 54,609 |  | 29,441 | 53.91% | 21,848 | 40.01% | 3,320 | 6.08% |
| Elixhauser comorbidities: n, % |  |  |  |  |  |  |  |  |
| … Congestive heart failure | 933 | 1.7% | 543 | 1.8% | 302 | 1.4% | 88 | 2.7% |
| … Cardiac arrhythmias | 1,622 | 3.0% | 907 | 3.1% | 547 | 2.5% | 168 | 5.1% |
| … Valvular disease | 360 | 0.7% | 193 | 0.7% | 118 | 0.5% | 49 | 1.5% |
| … Pulmonary circulation disorders | 76 | 0.1% | 46 | 0.2% | 21 | 0.1% | 9 | 0.3% |
| … Peripheral vascular disorders | 598 | 1.1% | 361 | 1.2% | 188 | 0.9% | 49 | 1.5% |
| … Hypertension, uncomplicated | 13,613 | 24.9% | 7,115 | 24.2% | 5,355 | 24.5% | 1,143 | 34.4% |
| … Hypertension, complicated | 557 | 1.0% | 295 | 1.0% | 198 | 0.9% | 64 | 1.9% |
| … Paralysis | 535 | 1.0% | 264 | 0.9% | 179 | 0.8% | 92 | 2.8% |
| … Other neurological disorders | 694 | 1.3% | 380 | 1.3% | 234 | 1.1% | 80 | 2.4% |
| … Chronic pulmonary disease | 1,511 | 2.8% | 818 | 2.8% | 560 | 2.6% | 133 | 4.0% |
| … Diabetes, uncomplicated | 5,574 | 10.2% | 2,931 | 10.0% | 2,132 | 9.8% | 511 | 15.4% |
| … Diabetes, complicated | 733 | 1.3% | 421 | 1.4% | 240 | 1.1% | 72 | 2.2% |
| … Hypothyroidism | 1,899 | 3.5% | 979 | 3.3% | 758 | 3.5% | 162 | 4.9% |
| … Renal failure | 2,102 | 3.8% | 1,303 | 4.4% | 556 | 2.5% | 243 | 7.3% |
| … Liver disease | 477 | 0.9% | 298 | 1.0% | 140 | 0.6% | 39 | 1.2% |
| … Peptic ulcer disease excluding bleeding | *Excluded due to less than 50 observations* | | | | | | | |
| … AIDS/HIV | *Excluded due to less than 50 observations* | | | | | | | |
| … Lymphoma | 52 | 0.1% | 31 | 0.1% | 16 | 0.1% | 5 | 0.2% |
| … Metastatic cancer | 103 | 0.2% | 64 | 0.2% | 31 | 0.1% | 8 | 0.2% |
| … Solid tumor without metastasis | 1,100 | 2.0% | 662 | 2.2% | 344 | 1.6% | 94 | 2.8% |
| … Rheumatoid arthritis/ collagen vascular diseases | 275 | 0.5% | 141 | 0.5% | 113 | 0.5% | 21 | 0.6% |
| … Coagulopathy | 448 | 0.8% | 275 | 0.9% | 105 | 0.5% | 68 | 2.0% |
| … Obesity | 3,961 | 7.3% | 2,101 | 7.1% | 1,478 | 6.8% | 382 | 11.5% |
| … Weight loss | 80 | 0.1% | 48 | 0.2% | 26 | 0.1% | 6 | 0.2% |
| … Fluid and electrolyte disorders | 1,752 | 3.2% | 1,033 | 3.5% | 399 | 1.8% | 320 | 9.6% |
| … Blood loss anemia | *Excluded due to less than 50 observations* | | | | | | | |
| … Deficiency anemia | 141 | 0.3% | 75 | 0.3% | 41 | 0.2% | 25 | 0.8% |
| … Alcohol abuse | 195 | 0.4% | 98 | 0.3% | 75 | 0.3% | 22 | 0.7% |
| … Drug abuse | 93 | 0.2% | 62 | 0.2% | 21 | 0.1% | 10 | 0.3% |
| … Psychoses | 89 | 0.2% | 40 | 0.1% | 39 | 0.2% | 10 | 0.3% |
| … Depression | 716 | 1.3% | 377 | 1.3% | 278 | 1.3% | 61 | 1.8% |
| Abbreviations: URS=Ureteroscopy; SWL=Extracorporeal shockwave lithotripsy; PCNL=Percutaneous nephrolithotomy; SD=Standard deviation | | | | | | | | |

**Supplementary table 3**: Association of index treatment and the number of re-interventions per patient

| **Dependent variable** | **OR** | **p-value** | **95% CI** | |
| --- | --- | --- | --- | --- |
| Index treatment (reference: URS) |  |  |  | |
| … PCNL | 1.330 | <.0001 | 1.22-1.46 | |
| … SWL | 1.494 | <.0001 | 1.42-1.57 | |
| Age (per year) | 0.987 | <.0001 | 0.99-0.99 | |
| Sex: male (reference: Female) | 1.137 | <.0001 | 1.09-1.19 | |
| Setting of index treatment: Outpatient (reference: Inpatient) | 3.776 | <.0001 | 2.56-5.58 | |
| Abbreviations: OR = Odds ratio; CI=Confidence interval; URS=Ureteroscopy; SWL=Extracorporeal shockwave lithotripsy; PCNL=Percutaneous nephrolithotomy. Model was adjusted for Elixhauser comorbidities, index treatment ICD code and year of index treatment. | | | |  |

**Supplementary table 4**: Association of index treatment and time to re-interventions

|  | **Time to first event** | | | **Time to any event** | | | **Time to any event** | | |
| --- | --- | --- | --- | --- | --- | --- | --- | --- | --- |
| **Dependent variable** | **HR** | **p-value** | **95% CI** | **HR** | **p-value** | **95% CI** | **HR** | **p-value** | **95% CI** |
| Index treatment (reference: URS) |  |  |  |  |  |  |  |  |  |
| … PCNL | 1.383 | <.0001 | 1.27-1.51 | 1.328 | <.0001 | 1.24-1.43 | 1.317 | <.0001 | 1.22-1.43 |
| … SWL | 1.557 | <.0001 | 1.48-1.64 | 1.456 | <.0001 | 1.4-1.52 | 1.538 | <.0001 | 1.47-1.61 |
| Treatment change after index treatment |  |  |  |  |  |  | 2.673 | <.0001 | 2.47-2.9 |
| Treatment change after index treatment * Index treatment PCNL |  |  |  |  |  |  | 0.651 | <.0001 | 0.55-0.77 |
| Treatment change after index treatment * Index treatment SWL |  |  |  |  |  |  | 0.513 | <.0001 | 0.46-0.57 |
| Age (per year) | 0.989 | <.0001 | 0.99-0.99 | 0.988 | <.0001 | 0.99-0.99 | 0.988 | <.0001 | 0.99-0.99 |
| Sex: male (reference: Female) | 1.155 | <.0001 | 1.11-1.2 | 1.138 | <.0001 | 1.1-1.18 | 1.135 | <.0001 | 1.1-1.17 |
| Setting of index treatment: Outpatient (reference: Inpatient) | 3.965 | <.0001 | 2.97-5.3 | 3.024 | <.0001 | 2.39-3.83 | 2.893 | <.0001 | 2.28-3.67 |
| Abbreviations: HR = Hazard ratio; CI=Confidence interval; URS=Ureteroscopy; SWL=Extracorporeal shockwave lithotripsy; PCNL=Percutaneous nephrolithotomy. Models were adjusted for Elixhauser comorbidities, index treatment ICD code and year of index treatment. | | | | | | | | | |

**Supplementary table 5**: Association of index treatment and urolithiasis-related health care costs

|  | **Costs for index treatment** | | | **Costs in total follow-up period** | | |
| --- | --- | --- | --- | --- | --- | --- |
| **Dependent variable** | **OR** | **p-value** | **95% CI** | **OR** | **p-value** | **95% CI** |
| Index treatment (reference: URS) |  |  |  |  |  |  |
| … PCNL | 2.069 | <.0001 | 2.04-2.10 | 1.941 | <.0001 | 1.9-1.99 |
| … SWL | 1.006 | 0.155 | 1.00-1.02 | 1.087 | <.0001 | 1.07-1.1 |
| Age (per year) | 1.000 | 0.665 | 1.00-1.00 | 0.997 | <.0001 | 1-1 |
| Sex: male (reference: Female) | 0.979 | <.0001 | 0.97-0.99 | 1.004 | 0.406 | 0.99-1.01 |
| Setting of index treatment: Outpatient (reference: Inpatient) | 0.288 | <.0001 | 0.26-0.32 | 0.984 | 0.802 | 0.86-1.12 |
| **Resulting costs [EUR]** | **AME** | **p-value** | **95% CI** | **AME** | **p-value** | **95% CI** |
| Index treatment |  |  |  |  |  |  |
| … URS | 1334 | <.0001 | 1193-1491 | 2979 | <.0001 | 2559-3469 |
| … PCNL | 2760 | <.0001 | 2467-3089 | 5783 | <.0001 | 4960-6744 |
| … SWL | 1342 | <.0001 | 1201-1501 | 3240 | <.0001 | 2781-3773 |
| Abbreviations: OR = Odds ratio; CI=Confidence interval; URS=Ureteroscopy; SWL=Extracorporeal shockwave lithotripsy; PCNL=Percutaneous nephrolithotomy. Models were adjusted for Elixhauser comorbidities, index treatment ICD code and year of index treatment. | | | | | | |

**Supplementary table 6**: Association of index treatment and urolithiasis-related sick leave days

|  | **Sick leave days after index treatment** | | | **Sick leave days in total follow-up period** | | | |  |  |
| --- | --- | --- | --- | --- | --- | --- | --- | --- | --- |
| **Dependent variable** | **OR** | **p-value** | **95% CI** | | **OR** | **p-value** | **95% CI** | |  |
| Index treatment (reference: URS) |  |  |  | |  |  |  | |  |
| … PCNL | 1.805 | <.0001 | 1.62-2.01 | | 1.827 | <.0001 | 1.64-2.04 | |  |
| … SWL | 1.311 | <.0001 | 1.24-1.39 | | 1.423 | <.0001 | 1.34-1.51 | |  |
| Age (per year) | 0.922 | <.0001 | 0.92-0.92 | | 0.916 | <.0001 | 0.91-0.92 | |  |
| Sex: male (reference: Female) | 1.791 | <.0001 | 1.70-1.88 | | 1.838 | <.0001 | 1.75-1.93 | |  |
| Setting of index treatment: Outpatient (reference: Inpatient) | 0.699 | 0.258 | 0.38-1.30 | | 0.834 | 0.572 | 0.44-1.56 | |  |
| **Resulting sick leave days [days]** | **AME** | **p-value** | **95% CI** | | **AME** | **p-value** | **95% CI** | |  |
| Index treatment |  |  |  | |  |  |  | |  |
| … URS | 5.3 | <.0001 | 3.90-7.20 | | 6.8 | <.0001 | 5.00-9.40 | |  |
| … PCNL | 9.8 | <.0001 | 7.10-13.5 | | 13.0 | <.0001 | 9.40-18.1 | |  |
| … SWL | 7.1 | <.0001 | 5.20-9.70 | | 10.1 | <.0001 | 7.40-13.8 | |  |
| Abbreviations: OR = Odds ratio; CI=Confidence interval; URS=Ureteroscopy; SWL=Extracorporeal shockwave lithotripsy; PCNL=Percutaneous nephrolithotomy. Models were adjusted for Elixhauser comorbidities, index treatment ICD code and year of index treatment. | | | | | | | | | |

**Supplementary figure 1**: Estimated risk scores for re-interventions by index treatment with or without subsequent treatment change


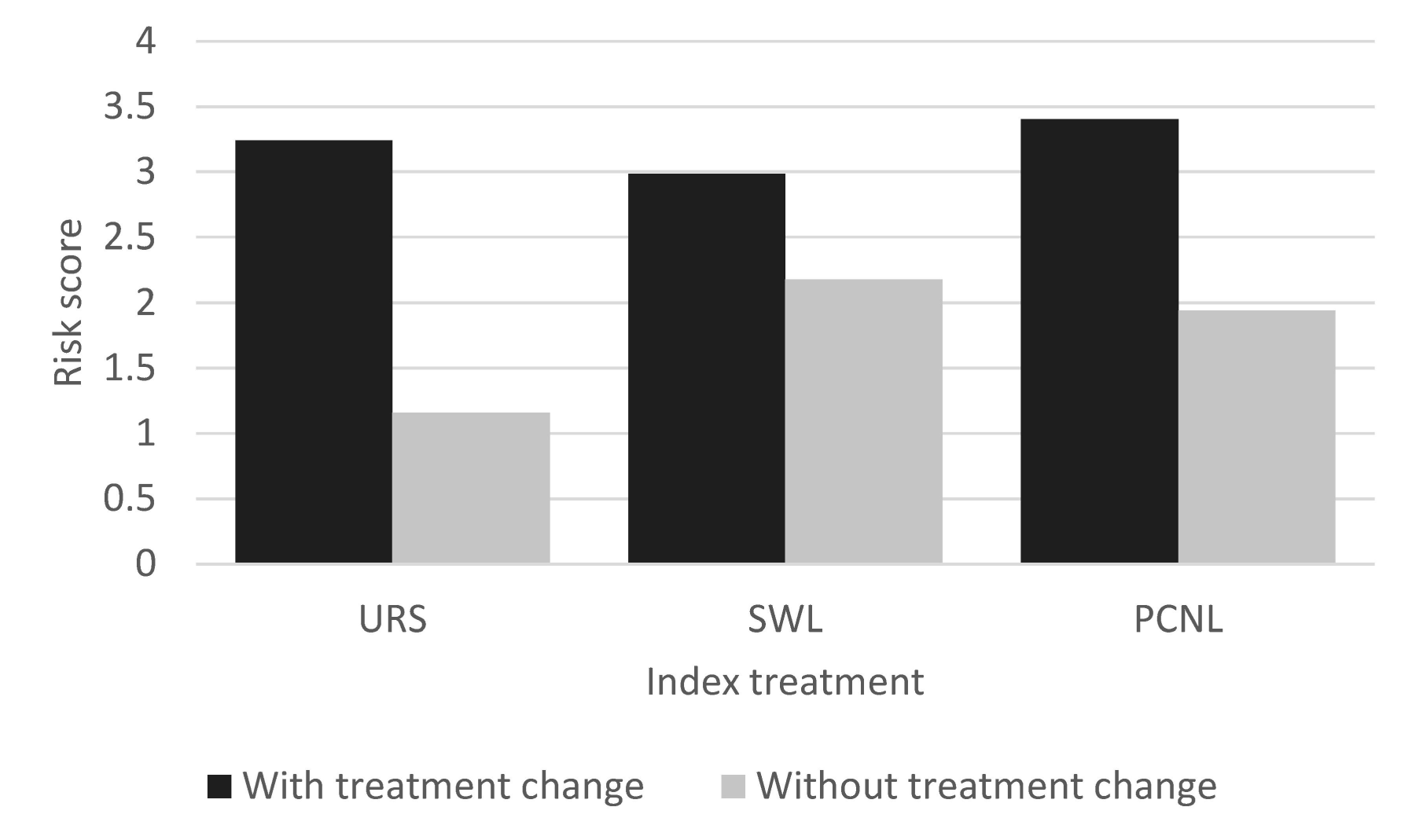

Supplement: Supplementary file 1 — Supplementary file1 (DOCX 105 KB) [file 345_2022_4180_MOESM1_ESM.docx]
